# iCoverT: A rich data source on the incidence of child maltreatment over time in England and Wales

**DOI:** 10.1371/journal.pone.0201223

**Published:** 2018-08-27

**Authors:** Michelle Degli Esposti, Jonathan Taylor, David K. Humphreys, Lucy Bowes

**Affiliations:** 1 Department of Experimental Psychology, University of Oxford, Oxford, United Kingdom; 2 Faculty of History, University of Oxford, Oxford, United Kingdom; 3 Department of Social Policy and Intervention, University of Oxford, Oxford, United Kingdom; Stellenbosch University, SOUTH AFRICA

## Abstract

Child maltreatment is a major public health problem, which is plagued with research challenges. Good epidemiological data can help to establish the nature and scope of past and present child maltreatment, and monitor its progress going forward. However, high quality data sources are currently lacking for England and Wales. We employed systematic methodology to harness pre-existing datasets (including non-digitalised datasets) and develop a rich data source on the incidence of Child maltreatment over Time (iCoverT) in England and Wales. The iCoverT consists of six databases and accompanying data documentation: Child Protection Statistics, Children In Care Statistics, Criminal Statistics, Homicide Index, Mortality Statistics and NSPCC Statistics. Each database is a unique indicator of child maltreatment incidence with 272 data variables in total. The databases span from 1858 to 2016 and therefore extends current data sources by over 80 years. We present a proof-of-principle analysis of a subset of the data to show how time series methods may be used to address key research challenges. This example demonstrates the utility of iCoverT and indicates that it will prove to be a valuable data source for researchers, clinicians and policy-makers concerned with child maltreatment. The iCoverT is freely available at the Open Science Framework (osf.io/cf7mv).

## Introduction

Child maltreatment is a major public health and social welfare problem world-wide [1,2]. However, child maltreatment research is plagued with challenges; from difficulties in measuring and establishing the scope of the problem, to ethical and practical obstacles hindering the use of randomised controlled trials to determine the effectiveness of interventions [3,4].

Good epidemiological data are needed to overcome such research challenges, in particular regularly collected national incidence data [1]. These data can help characterise the nature and extent of child maltreatment, and coupled with advanced statistical methods can also be used to evaluate the effect of planned or unplanned interventions [5,6]. Exploring long-term trends and changes to the incidence of child maltreatment can shed new insights on this emergent and complex social health issue, providing directly translatable evidence for practitioners and policy-makers.

Countries are beginning to respond to this need by routinely collecting national estimates of the incidence of child maltreatment. The United States, Canada and the Netherlands have developed professional surveys to prospectively collect incidence estimates [7–9], whereas England and Wales extract administrative data. Following the introduction of the Children Act 1989, England and Wales have routinely collected administrative data on the number of children referred to, and assessed by, social services [10]. These data have been compiled and published on a national database since 2008 under the revised children in need census [11]. Although these data go some way in providing national incidence data on child maltreatment, considerable progress is still needed before there are sufficient data to investigate long-term trends and to implement robust statistical techniques, such as time series analyses. More comprehensive usable data sources are needed to advance current research and understanding.

To address this need, we developed a rich epidemiological data source on the incidence of child maltreatment over Time (iCoverT) in England and Wales by harnessing pre-existing datasets. The iCoverT offers new opportunities to quantify temporal trends and changes over time, whilst also providing a valuable public health surveillance tool for monitoring child maltreatment.

## Methods

### Overview

**Fig 1:**
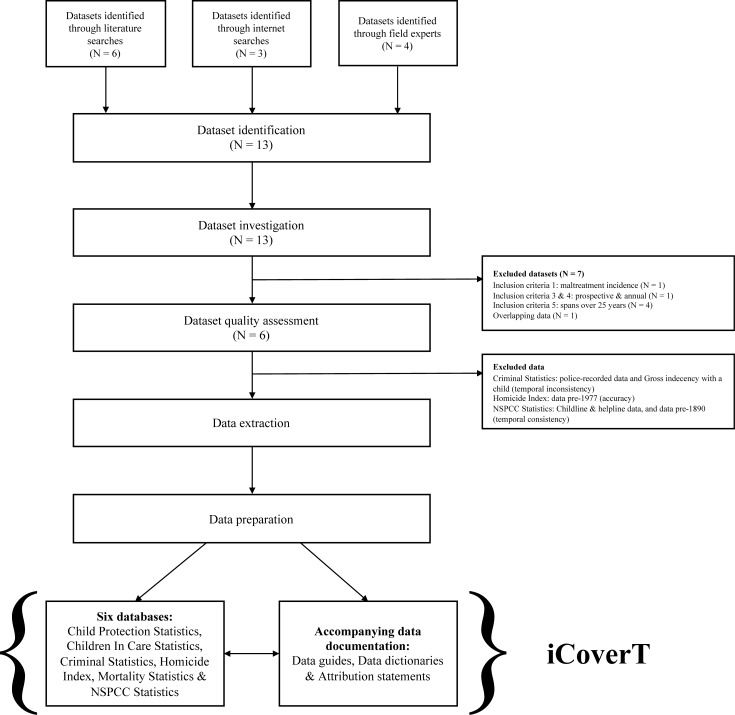
Schematic diagram illustrating the process of developing the iCoverT.

We adapted systematic review and routinely-collected data recommendations from the PRISMA and RECORD statements to devise a systematic strategy for identifying, investigating and assessing pre-existing datasets of routinely collected data [12,13]. Ethical approval was not required because all data were fully anonymised and publicly available before we accessed them. Out of 13 identified datasets, data from six datasets were extracted and prepared as six temporally consistent databases. These six databases on the incidence of child maltreatment over time and their accompanying documentation form the iCoverT (Fig 1). The iCoverT is a freely accessible data source and new data may be deposited to maintain and update it [14].

### Dataset identification

Three main search strategies were used to identify relevant datasets on the incidence of child maltreatment in England and Wales, including literature and internet searches using historically sensitive search terms and contact with experts within academia and health provision. We searched for datasets which met the following inclusion criteria: (1) data could be used to estimate the incidence of child maltreatment. Child maltreatment was defined as one, or a combination of, physical abuse, neglect, sexual abuse and emotional maltreatment to a child aged 18 years old or under. Bullying by peers and witnessing intimate partner violence were not included in this definition; (2) data were nationally representative of England and Wales, including or excluding Northern Ireland; (3) data were collected prospectively; (4) data were collected annually; and (5) data were collected and available for a period of at least 25 years. If data were overlapping only the richest dataset was included. We identified 13 relevant datasets; six datasets were identified from literature searches, three from internet searches, and four from field experts (Fig 1).

### Dataset investigation and quality assessment

We investigated the 13 identified datasets by asking the following key questions: who collects the data, why are the data collected, when were the data collected, what data are collected, and where are the data located. Answers for each dataset were obtained by reading accompanying dataset information and contacting the authorities responsible for collecting, publishing and/or holding the data. We made 23 enquiries to different government departments, organisations, libraries and online data repositories, submitted nine submitted Freedom of Information (FOI) requests, and visited seven libraries and archives. Following these investigations, seven datasets were excluded because they did not satisfy the inclusion criteria (S1 Table), leaving the six included datasets: Child Protection Statistics, Children In Care Statistics, Criminal Statistics, Homicide Index, Mortality Statistics and National Society for the Prevention of Cruelty to Children (NSPCC) Statistics.

We assessed the quality of the six remaining datasets using an adapted quality assessment tool [15–17]. In line with our pre-specified rating guidance, we judged data quality to be *good*, *satisfactory* or *problematic* for eight quality criteria (representativeness, missingness, accuracy, temporal consistency, validity, definitions, timeliness, interpretability) (S2 Table). If a dataset was rated problematic on a criterion we explored feasible strategies to address the problem. Datasets were rated satisfactory to good on the majority of criteria, except all datasets were rated to have problematic temporal consistency. In addition, Children In Care Statistics showed problematic missingness and accuracy, Child Protection Statistics had problematic accuracy, and Criminal Statistics demonstrated problematic interpretability (S2B Table). Although no dataset was fully excluded due to unresolvable quality issues, four data variables were excluded due to unresolvable temporal consistency problems, and two were truncated due to unresolvable temporal consistency and accuracy problems (S3 Table).

### Data extraction

We located, saved and indexed all available data for the six included datasets using the online shareable reference manager Zotero. Data were found in various formats both within and across datasets. Data available electronically or via websites were saved directly to Zotero, whilst data only available as hardcopies at libraries and archives were photographed, uploaded, and indexed accordingly. Data extraction depended on the format of the original data and their sources. Data available as hardcopies or embedded within PDFs required manual extraction, whilst some data in excel or common separated value (CSV) formats were electronically copied across. Most data required year-by-year extraction from separate sources, whilst some data were part-collated across years (e.g. 5-year period). M.D.E. systematically extracted all data, entering the data for each dataset into separate standardised CSV templates to generate six databases.

The reliability of the data extraction process and accuracy of extracted data were checked by embedding internal calculations within the databases to validate the relationships between data variables. For example, for the Children In Care Statistics database “Males in care in England and Wales” was checked to equal the sum of “Males in care in England” and “Males in care in Wales”. In addition, a second data extractor (J.T.) carried out independent data checks of 100% of the data. The average interrater agreement across databases was 99.1% and any discrepancies were discussed and resolved by consensus. Data were further visualised to identify any potential anomalies.

### Data preparation

Following data extraction, we prepared the data in two main stages to generate six databases of complete, temporally consistent time series data.

#### Stage 1: Generating temporally consistent data

Our quality assessment identified that all six databases suffered from temporal inconsistency problems due to various changes over time. These changes ranged from structural and organisational, to data and terminology. We investigated all changes over time and implemented appropriate data linkage strategies to address them. We employed five main strategies: (a) derived calculations to address temporal changes and generate equivalent data across years; (b) adopted broader categories to obtain consistent data variables across time; (c) identified and matched equivalent items over time; (d) consistently prioritised specific data sources; and (e) determined the change to have no substantial effect on the data. Full details are specified in S4 Table. Despite addressing all temporal inconsistencies, not all strategies may comprehensively account for changes over time. We therefore recommend the inclusion of dummy variables in data analyses for specific changes, where dummy variables may be coded to represent these changes over time. Our recommendations are indicated in S4 Table.

#### Stage 2: Adjusting the data

We carried out data adjustments if: more than 20% of the data was missing, there were data idiosyncrasies, data did not cover a 12-month period, and data were inconsistently rounded. The Criminal Statistics and the Homicide Index databases did not require any data adjustments. Only one data variable from the Children In Care Statistics database was found to have more than 20% missing data, which was addressed using ad-hoc missing data imputations. Child Protection Statistics, Children In Care Statistics, Mortality Statistics and NSPCC Statistics showed specific idiosyncrasies within their data. Each idiosyncrasy was minor and easily addressed. One year within the Children In Care Statistics database and two years within the NSPCC Statistics database did not cover the full 12-month period. For these three years, data were adjusted to account for missing months. Child Protection Statistics and Children In Care Statistics data were inconsistently rounded over time. As a result, data variables were rounded to the same level of precision. Missing data imputations and data adjustments are described in S5 Table.

### Data documentation

We systematically and comprehensively documented all stages of dataset investigations and assessment, and data extraction and preparation. For each database, these details were synthesised and prepared as three types of data documentation: Data guides, Data dictionaries and Attribution statements. Data guides include background information obtained from dataset investigations, and where appropriate, more detailed information about data variables (context and definitions). Data dictionaries describe the contents, format, and structure of each database. Attribution statements document in full all data sources and corresponding permissions.

## Results and discussion

### Description of the iCoverT

#### iCoverT structure

The iCoverT is a comprehensive data source on the incidence of child maltreatment over time [14]. It consists of six separate databases, which are each accompanied by a set of three data documents (Data guide, Data dictionary, Attribution statement). An overview of the iCoverT is detailed in machine-actionable and readable Data Documentation Initiative (DDI) compliant metadata (XML and PDF format).

#### Database characteristics

Characteristics of the iCoverT databases are summarised in Table 1. Child Protection Statistics, Children In Care Statistics, Criminal Statistics, Homicide Index, Mortality Statistics and NSPCC Statistics are all forms of administrative data, which have been annually collected over time. For all databases, data were first collected by local authorities or regional branches and then aggregated, checked and validated by centralised departments or headquarters to form national estimates. Centralised departments and headquarters include various government departments, from the Office for National Statistics to the Department of Health, and the NSPCC’s London Headquarters.

**Table 1:** Summary of database characteristics.

Database	Data sources (permission)^a^	Population	Geographical coverage	Time range (intervals)	Data variables		Data	
					Number	Additional information	Type	Precision
Child Protection Statistics	Children and young persons on the child protection register, England, Department of Health; Child protection register statistics for Wales, Welsh Government; Children in need in England & Wales, Department for Education & Welsh Government (OGL)	Children on the child protection register	England & Wales	1989–2016 (annual)	100	• Country • Child’s gender • Child’s age • Category of abuse • Children on the register or new registrations	Count	Nearest 5, 10 or 100
Children In Care Statistics	Children in care in England and Wales, Department for Health; Children looked after in England, Department for Education; Children in care of local authorities in Wales, Welsh Office (OGL)	Children in the care of local authorities	England & Wales	1949–2016 (annual)	37	• Country • Child’s gender • Child’s age • Children in care or children entering care	Count	Nearest 10 or 100
Criminal Statistics	Criminal statistics in England and Wales, Home Office & Ministry of Justice (OGL)	Persons accused of offences against children	England & Wales	1893–2016 (annual)	15	• Offence type • Person accused or found guilty	Count	Integer
Homicide Index	Homicides in England and Wales, Home Office & Office for National Statistics (OGL)	Police-recorded child homicides	England & Wales	1977–2016 (annual)	24	• Child’s gender • Child’s age	Count	Integer
Mortality Statistics	20^th^ and 21^st^ century mortality datasets, England and Wales, Office for National Statistics (OGL)	Child deaths caused by homicide, unknown causes and injury from undetermined intent	England & Wales	1858–2016 (annual)	73	• Child’s gender • Child’s age • Cause of death	Count	Integer
NSPCC Statistics	Annual reports, NSPCC (NSPCC written permission under OGL)	Children involved in NSPCC cases	England, Wales & Northern Ireland	1890–1985 (annual)	23	• NSPCC cases or children involved • Child’s gender • Child’s age • Reason for NSPCC involvement • Who referred the case to the NSPCC	Count	Integer

^a^ Full details data sources and permissions are documented in Attribution statements.

OGL **=** Open Government License.

Five databases represent the number of victims of child maltreatment, be it children on the child protection register or child homicide victims, whereas one database represents the number of perpetrators of child maltreatment (Criminal Statistics). All databases cover England and Wales, with data from NSPCC Statistics also covering Northern Ireland. The average time range across databases is just under 90 years. Mortality Statistics is the oldest database collected since 1858 while Child Protection Statistics is the newest database first collected in 1988. Although we excluded more recent data from NSPCC Statistics due to temporal inconsistencies, all databases continue to be collected today.

#### Descriptive statistics

In total, there are 272 data variables. These data variables include detailed information on the gender and age of victim, as well as information about the type of maltreatment, who reported the incident, and the country (e.g. England, Wales). All data are freely available online, and Table 2 shows descriptive statistics for main data variables on the overall number of child maltreatment incidents, and the victims’ gender. All data are counts and range from 0 to 15,9407 depending on the database, data variable and year. Across databases, there is high degree of variance within data variables due to considerable year-on-year fluctuations.

**Table 2:** Descriptive statistics of main data variables.

Database	Main data variables	Time range	Observations	Missing observations	Mean (sd)	Min	Max
Child Protection Statistics^a^	Children^b^ on the child protection register in England and Wales	1988–2016	29	0	37989.66 (7988.67)	27700	53400
	Males^b^ on the child protection register in England and Wales	1988–2016	27	2	18685.19 (3689.77)	14300	26300
	Females^b^ on the child protection register in England and Wales	1988–2016	27	2	18222.22 (3830.48)	13200	25200
Children In Care Statistics^c^	Children^b^ in care in England and Wales	1949–2016	65	2	71455.38 (13493.53)	52100	101200
	Males^b^ in care in England and Wales	1952–2016	61	3	40914.75 (9273.98)	27900	61000
	Females^b^ in care in England and Wales	1952–2016	61	3	31424.59 (4235.43)	24300	41100
Criminal Statistics	Persons^d^ guilty of Cruelty to children	1893–2016	113	11	1040.12 (964.31)	89	3450
	Persons^d^ guilty of Infanticide	1923–2016	87	7	9.74 (7.73)	0	39
	Persons^d^ guilty of Abandoning a child under 2	1893–2016	113	11	2.43 (2.20)	0	10
	Persons^d^ guilty of Unlawful sex with under 13s	1893–2016	112	12	68.88 (26.06)	17	136
	Persons^d^ guilty of Unlawful sex with 13 to 16 year olds	1893–2016	112	12	302.02 (221.36)	34	792
Homicide Index	Child homicide victims^e^	1977–2016	40	0	69.63 (15.41)	38	100
	Male homicide victims^e^	1977–2016	40	0	37.73 (9.65)	18	61
	Female homicide victims^e^	1977–2016	40	0	31.85 (8.98)	18	56
Mortality Statistics	Child^f^ deaths caused by homicide	1858–2016	159	0	99.61 (55.26)	19	236
	Male^f^ deaths caused by homicide	1858–2016	159	0	50.57 (28.63)	8	123
	Female^f^ deaths caused by homicide	1858–2016	159	0	49.04 (27.52)	9	123
	Child^f^ deaths caused by unknown causes	1858–2016	159	0	3470.24 (7426.14)	5	26179
	Male^f^ deaths caused by unknown causes	1858–2016	159	0	1910.92 (4071.62)	3	14202
	Female^f^ deaths caused by unknown causes	1858–2016	159	0	1559.32 (3355.31)	2	1197
	Child^f^ deaths caused by injury of undetermined intent	1968–2016	49	0	49.10 (14.91)	26	91
	Male^f^ deaths caused by injury of undetermined intent	1968–2016	49	0	29.71 (9.34)	14	60
	Female^f^ deaths caused by injury of undetermined intent	1968–2016	49	0	19.39 (6.91)	7	38
NSPCC Statistics^g^	Children^b^ involved in NSPCC cases	1890–1985	96	0	92142.35 (34266.04)	7463	159407
	Males^b^ involved in NSPCC cases	1940–1969	30	0	52896.97 (4384.12)	42022	62440
	Females^b^ involved in NSPCC cases	1940–1969	30	0	50351.53 (4243.15)	39588	59125

^a^ The number of males and females on the child protection register do not add up to the total number of children as registrations were for unborn children and/or details were unknown (from approximately 0% to 3% depending on the year). In addition, some figures were reported to the nearest 100 (see Table 1) and this resulted in rounding error.

^b^ Aged under 18 years old.

^c^ The number of males and females do not add up to the total number of children in care as some figures were rounded to the nearest 100 (see Table 1) and this resulted in rounding error.

^d^ Aged over 15 years old.

^e^ Aged under 16 years old at time of death.

^f^ Aged under 15 years old at time of death.

^g^ The number of males and females do not add up to the total number of children involved in NSPCC cases as the data were not collected for the same periods of time.

### Utility of the iCoverT

The iCoverT is a rich epidemiological data source, which extends current maltreatment incidence data in England and Wales by over 80 years [11,18–21]. As a result, the iCoverT offers the statistical power to use advanced statistical methods, such as fitting regression models, interrupted time series designs, and ARIMA modelling. The databases are also well-suited to help answer key issues within child maltreatment research. The historical data can shed light on past maltreatment and help to contextualise current maltreatment, whilst more recent data can be used to monitor current and future rates of child maltreatment. The iCoverT may also be used to evaluate the impact of planned or unplanned interventions on the occurrence of child maltreatment (e.g. specific child protection efforts).

### Proof-of-principle analysis

To demonstrate the potential utility of the iCoverT, we carried out a proof-of-principle analysis on a subset of its data taken from the Criminal Statistics database. To date, there are contradicting findings on whether trends in child maltreatment are decreasing. Some scientific evidence indicates decreasing trends since the 1970s and other evidence suggests no significant change over time [19,20]. However, to our knowledge, no empirical evidence quantitatively investigates maltreatment trends in England and Wales before the 1970s. We therefore addressed the question: Has the incidence of guilty convictions for the criminal offence *Cruelty to children* decreased from 1893 to 1970?

**Fig 2:**
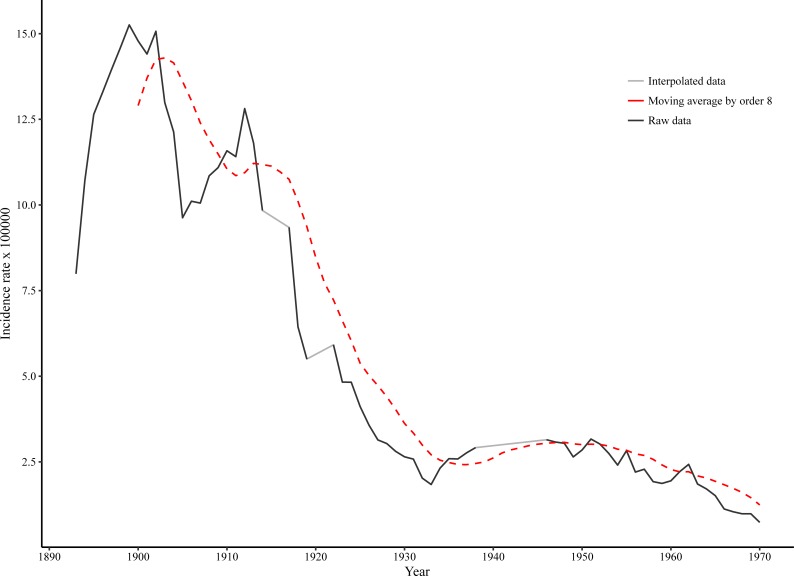
Persons guilty of Cruelty to children from 1893 to 1970. The graph plots the raw data *(black)*, interpolated data *(grey)*, and an eight-year moving average *(red)* of the age-standardised incidence (per 1000,000) of persons found guilty of the criminal offence, *Cruelty to children*, by calendar year.

All statistical analyses were carried out in R (version 3.4.1). Age-standardised incidence rates (guilty persons per 100,000 population) were calculated from the number of persons found guilty of *Cruelty to children* and the total population of persons aged over 15 [22]. There were missing data due to disruptions caused by the First and Second World Wars (1915, 1916, 1920, 1921 and 1939–1945). These missing values were imputed using linear interpolation for non-seasonal univariate time series. We derived and plotted an eight-year moving average as this best visualised the data, smoothing short-term fluctuations and isolating the long-term trend (Fig 2). The smoothed time series shown in Fig 2 illustrates an overall downward trend in incidence rates of persons found guilty of *Cruelty to children* from 1900 to 1970. This decline is particularly steep between 1900 and 1930, plateauing out to a gentler decline from 1940 to 1970. Although this may suggest that the incidence of the criminal offence *Cruelty to children* fell between 1893 and 1970, a more careful historical analysis is needed to establish whether this trend reflects real decreases in the number of maltreatment-related crimes or whether it reflects wider changes to the judicial system, such as stricter guidelines on determining guilt. Nonetheless, this finding provides historical perspective and sheds light on longer-term, as well as more recent, maltreatment trends. This example also shows that the iCoverT can be analysed using time series methods to help answer key questions within child maltreatment research.

### Strengths of the iCoverT

The iCoverT has a number of strengths. The first strength lies in the systematic and exhaustive methods used to develop each database and their accompanying data documentation. All identified datasets underwent thorough investigations and were assessed against pre-specified inclusion and quality criteria. Data were manually extracted and checked in full by a second extractor. Despite complexities to the data extraction process, there was good agreement between extractors (99.1%) and further checks were carried out to ensure the accuracy of the data. In addition, we included unique steps to generate complete, temporally consistent data, applying data linkage strategies and data adjustments where appropriate. All stages of dataset identification, investigation, assessment, data extraction and preparation were meticulously documented.

The second strength lies in the richness of the data. The iCoverT consists of six databases with 272 data variables and spans from 1858 to 2016. The databases extend current data by over 80 years, and each database may shed a different light on the complex construct of child maltreatment. For example, NSPCC Statistics may capture more types of child maltreatment than Mortality Statistics, which only captures severe cases of physical abuse and/or neglect. Data variables may also offer important insights as they provide additional breakdowns, including information on: country, gender and age of victims, and type of child maltreatment. Third, the databases are nationally representative of England and Wales. Research on this type of epidemiological data results in robust and translatable national-level evidence, which is most pertinent for informing public health polices and interventions. Lastly, because new relevant data will become available each year, the iCoverT has potential for maintenance and growth. We intend to continue to collect data going forward, regularly updating and maintaining the iCoverT as a freely accessible data source for research.

### Limitations of the iCoverT

The iCoverT also has several limitations. First, most data were not originally collected for research purposes. Although we exhaustively investigated and addressed all identified confounders, the data may be influenced by obscure operational processes and changing organisation priorities that we are unaware of [23]. Second, while all data were reported annually, this temporal information may not necessarily reflect the occurrence of maltreatment in that specific year. This is particularly relevant for Mortality Statistics as previous research highlights a reporting delay in registering deaths [19]. However, the most recent release relating to Mortality Statistics states that the median registration is only 5 days, which would minimally effect the temporal accuracy of these data [24]. Third, we identified the relevant dataset of Hospital Episode Statistics for Admitted Patient Care in England [25]. Despite extracting relevant data from 1999 to 2016 and identifying earlier data from 1980 to 1998, we were unable to include this potentially valuable dataset due to financial costs (NHS Digital quoted a minimum cost of £1800 which exceeded available funds).

## Conclusions

The iCoverT is a rich, freely accessible data source on the incidence of child maltreatment over time in England and Wales. The development of the iCoverT, as described in this article, demonstrates how systematic methods can be used to overcome practical obstacles and harness pre-existing datasets from across disciplines. We believe that the iCoverT will be an invaluable data source and public health surveillance tool for researchers, clinicians and policy-makers concerned with child maltreatment.

## Supporting information

S1 Table Excluded datasets and reason(s) for exclusion. (DOCX)

S2 Table Quality assessment tool for included datasets (S2A Table) and quality assessment of included datasets (S2B Table). (DOCX)

S3 Table Excluded and truncated data variables with reason(s) for exclusion. (DOCX)

S4 Table Summary of changes over time, data linkage strategies and dummy variable recommendations. (DOCX)

S5 Table Summary of data adjustments. Note that full details of data adjustments for each database are detailed in the Data dictionaries.
(DOCX)

## References

[1] Butchart A, Phinney Harvey A, Kahane T, Mian M, Furniss T. Preventing child maltreatment: guide to action and generating evidence. WHO 2006 Available from: http://www.who.int/violence_injury_prevention/publications/violence/child_maltreatment/en/

[2] Gilbert R, Kemp A, Thoburn J, Sidebotham P, Radford L, Glaser D, et al Recognising and responding to child maltreatment. Lancet. 2009;373: 167–180. 10.1016/S0140-6736(08)61707-9 19056119

[3] Milling Kinard E. Methodological and practical problems in conducting research on maltreated children. Child Abuse Negl. 1994;18: 645–656. 7953904 10.1016/0145-2134(94)90014-0

[4] Fallon B, Trocmé N, Fluke J, MacLaurin B, Tonmyr L, Yuan YY. Methodological challenges in measuring child maltreatment. Child Abus Negl. 2010;34: 70–79. 10.1016/j.chiabu.2009.08.008 20053453

[5] Hyndman RJ, Athanasopoulos G. Forecasting: principles and practice. OTexts; 2014.

[6] Jud A, Fegert JM, Finkelhor D. On the incidence and prevalence of child maltreatment: A research agenda. Child Adolesc Psychiatry Ment Health. 2016;10 10.1186/s13034-016-0105-8 27303442 PMC4907083

[7] Trocmé NM, MacLaurin BJ, Fallon BA, Daciuk JF, Tourigny M, Billingsley DA. Canadian incidence study of reported child abuse and neglect: Methodology. Can J Public Health. 2001;92: 259–63. 11962109 10.1007/BF03404956 PMC6979859

[8] Sedlak AJ, Broadhurst DD, Westat. Executive Summary of the Third National Incidence Study of Child Abuse & Neglect (NIS-3). National Center on Child Abuse and Neglect (DHHS) Washington, DC; 1996.

[9] Euser S, Alink LRA, Pannebakker F, Vogels T, Bakermans-Kranenburg MJ, Van IJzendoorn MH. The prevalence of child maltreatment in the Netherlands across a 5-year period. Child Abuse Negl. 2013;37: 841–851. 10.1016/j.chiabu.2013.07.004 23938018

[10] HMSO. Children Act 1989. Family Law 1989.

[11] Munro ER, Brown R, Manful E. Safeguarding children statistics: the availability and comparability of data in the UK. London; 2011.

[12] Benchimol EI, Smeeth L, Guttmann A, Harron K, Moher D, Peteresen I, et al The REporting of studies Conducted using Observational Routinely-collected health Data (RECORD) Statement. PLoS Med. 2015;12: 1–22. 10.1371/journal.pmed.1001885 26440803 PMC4595218

[13] Moher D, Liberati A, Tetzlaff J, Altman DG. Preferred Reporting Items for Systematic Reviews and Meta-Analyses: The PRISMA Statement. PLoS Med. 2009;6: e1000097 10.1371/journal.pmed.1000097 19621072 PMC2707599

[14] Degli Esposti M, Taylor J, Bowes L, Humphreys DK. iCoverT: A data source on the incidence of Child maltreatment over Time in England and Wales; 2018 [cited 2018 Jul 16]. Database: Open Science Framework [Internet]. doi: 10.17605/OSF.IO/CF7MV 10.1371/journal.pone.0201223 PMC6110478 30148834

[15] Bain MRS, Chalmers JWT, Brewster DH. Routinely collected data in national and regional databases—an under-used resource. J Public Health. 1997;19: 413–418. 10.1093/oxfordjournals.pubmed.a024670 9467147

[16] Rao C, Lopez AD, Yang G, Begg S, Ma J. Evaluating national cause-of-death statistics: principles and application to the case of China. Bull World Health Organ. 2005;83: 618–625. 16184281 PMC2626325

[17] Joubert J, Rao C, Bradshaw D, Vos T, Lopez AD. Evaluating the Quality of National Mortality Statistics from Civil Registration in South Africa, 1997–2007. PLoS One. 2013;8: e64592 10.1371/journal.pone.0064592 23724066 PMC3664567

[18] Bentley H, O’Hagen O, Brown A, Vasco N, Lynch C, Peppiate J, et al How safe are our children? The most comprehensive overview of child protection in the UK. NSPCC; 2016 Available from: https://www.nspcc.org.uk/globalassets/documents/research-reports/how-safe-children-2016-report.pdf

[19] Hardelid P, Davey J, Dattani N, Gilbert R. Child Deaths Due to Injury in the Four UK Countries: A Time Trends Study from 1980 to 2010. PLoS One. 2013;8: e68323 10.1371/journal.pone.0068323 23874585 PMC3707924

[20] Gilbert R, Fluke J, O’Donnell M, Gonzalez-Izquierdo A, Brownell M, Gulliver P, et al Child maltreatment: variation in trends and policies in six developed countries. Lancet. 2012;379: 758–772. 10.1016/S0140-6736(11)61087-8 22169108

[21] Sidebotham P, Atkins B, Hutton JL. Changes in rates of violent child deaths in England and Wales between 1974 and 2008: an analysis of national mortality data. Arch Dis Child. 2012;97: 193–199. 10.1136/adc.2010.207647 21525527

[22] Office for National Statistics. Population Estimates for UK, England and Wales, Scotland and Northern Ireland [Internet]. 2017 [cited 2017 Nov 28]. Available from: https://www.ons.gov.uk/peoplepopulationandcommunity/populationandmigration/populationestimates/datasets/populationestimatesforukenglandandwalesscotlandandnorthernireland

[23] Morgan R, Maguire M, Reiner R. The Oxford handbook of criminology. Oxford University Press; 2012.

[24] Office for National Statistics. Quarterly mortality report, England [Internet]. 2017 [cited 2018 Feb 10]. Available from: https://www.ons.gov.uk/peoplepopulationandcommunity/birthsdeathsandmarriages/deaths/articles/quarterlymortalityreports/quarter1jantomar2017

[25] NHS Digital. Hospital Admitted Patient Care Activity, 2016–17 [Internet]. 2017 [cited 2018 Feb 10]. Available from: https://digital.nhs.uk/catalogue/PUB30098

