# Supplementary material for: iCoverT: A rich data source on the incidence of child maltreatment over time in England and Wales
Source: PLoS One. 2018 Aug 27;13(8):e0201223. doi: 10.1371/journal.pone.0201223 (PMC6110478; doi:10.1371/journal.pone.0201223)
Supplement: S1 Table — (DOCX) [file pone.0201223.s001.docx]

**S1 Table. Excluded datasets and reason(s) for exclusion.**

| **Excluded dataset** | **Reason for exclusion** |
| --- | --- |
| Annual reports, Barnardo’s | **Inclusion criteria 1**  Contact with the Barnardo’s Archive and Administration Officer identified that their data did not definitively measure the number of children helped by Barnardo’s as the types of projects, services, and home run by Barnardo’s changed drastically. As a result, the data could not be used to estimate the incidence of child maltreatment |
| Child death reviews, England, Department for Education | **Inclusion criteria 5**  Data on child death reviews collected from local safeguarding children boards was first introduced in April 2008. As a result, the data did not cover a period of at least 25 years |
| Child sexual exploitation and abuse, National Strategic Assessment, National Crime Agency | **Inclusion criteria 5**  Data pertaining to child sexual exploitation and abuse were first published in 2014. As a result, the data did not cover a period of at least 25 years |
| Crime Survey England and Wales^a^, Office for National Statistics | **Inclusion criteria 5**  The two relevant indicators of child maltreatment incidence: “Victimisation of children aged 10 to 15” and “Abuse during childhood” were first collected from 2008/09 and 2014/15, respectively. As a result, the data did not cover a period of at least 25 years |
| Hospital Episode Statistics, Admitted patient care, England, Department of Health & NHS England | **Inclusion criteria 5**  “Admitted patient care - External causes" data were collected from 1989/90. However, the data were only published from 1998/99. A FOI request was placed with the Department of Health to obtain the earlier unpublished data from 1989/90 to 1997/98. This request, and subsequent appeal, were rejected under Section 12(1) of the FOI Act, where public authorities are not obliged to comply with a request for information if it estimates the cost of complying would exceed the appropriate limit. A subsequent Data Access Request application was placed with NHS digital. Although this application was approved, the minimum cost was estimated at £1800 (excluding VAT), which exceeded available funds. As a result, the unpublished data were not obtained, and the remaining published data did not cover a period of at least 25 years |
| Report on the work of the Prison Department, Statistical tables, Home Office | **Overlapping data**  Prison statistics overlapped with criminal statistics. Because prison statistics covered a shorter time period than criminal statistics (first published in 1923) this dataset was excluded |
| Child abuse and neglect in the UK today, NSPCC^b^ | **Inclusion criteria 3 & 4**  The data were retrospective self-report, which were collected on two separate occasions (1998/99 and 2009). The data were therefore not prospective or annually collected |

FOI = Freedom of Information.

^a^ Prior to 1 April 2012 the Crime Survey for England and Wales was known as the British Crime Survey and was published by the Home Office.

^b^ Radford L, Corral S, Bradley C, Fisher H, Bassett C, Howat N, Collishaw S. Child abuse and neglect in the UK today. London: NSPCC; 2011.
