# Supplementary material for: iCoverT: A rich data source on the incidence of child maltreatment over time in England and Wales
Source: PLoS One. 2018 Aug 27;13(8):e0201223. doi: 10.1371/journal.pone.0201223 (PMC6110478; doi:10.1371/journal.pone.0201223)
Supplement: S2 Table — (DOCX) [file pone.0201223.s002.docx]

**S2A Table. Quality assessment tool for included datasets.**

| **Quality criterion** | **Question** | **Rating** | **Rating guidance** |
| --- | --- | --- | --- |
| Representativeness | Are data representative of children who are maltreated across England and Wales? | Good | Data are nationally representative, capturing all ages (under 19 years), types (e.g., physical abuse, neglect, sexual abuse and emotional abuse) and severities of child maltreatment |
|  |  | Satisfactory | Data are nationally representative, if not comprehensive, and capture more than one age group, type and/or severity of child maltreatment |
|  |  | Problematic | Data are not nationally representative or only capture a specific subset of children who are maltreated in England and Wales. For example, deaths only from shaken baby syndrome or by Munchausen’s by proxy |
| Missingness | Are there missing data? | Good | Included variable(s) within the dataset are missing less than 5% of data items over time |
|  |  | Satisfactory | Included variable(s) are missing less than 20% of data items over time |
|  |  | Problematic | Included variable(s) are missing more than 20% of data items over time |
| Accuracy | Are the data consistently reported across publications? | Good | There is perfect agreement between different publications; the same figures are published for every data item |
|  |  | Satisfactory | There is good agreement between different publications, with over 80% of figures being consistent across publications |
|  |  | Problematic | There are data discrepancies between publications for over 80% of figures |
| Temporal consistency | Are the data measuring the same thing from year to year? | Good | There are no changes to the data, data format or data collection method over time |
|  |  | Satisfactory | Despite some basic changes to the data, data format or data collection method over time, the data may still be considered relative measure over and thus broadly temporally consistent |
|  |  | Problematic | Substantial changes to the data, data format or data collection method take place, making the raw data difficult to compare over time |
| Validity | Is the data collection method appropriate for measuring what it is meant to measure? | Good | The data collection method relies on suitable primary sources of information and follows a logical process of data collection and data aggregation, which is internally checked and validated |
|  |  | Satisfactory | The data collection method relies on suitable primary sources of information and follows a logical process of data collection, but is not formally checked or validated |
|  |  | Problematic | The data collection method does not use appropriate information and therefore there is no logical certainty that the data measure what they are said to measure |
| Definitions | Are data variables clearly defined? | Good | Clear definitions are provided for all variables |
|  |  | Satisfactory | Variables are defined or are clearly labelled |
|  |  | Problematic | No definitions are provided for the data variables and it is unclear what different variables are measuring |
| Timeliness | Are the data temporally precise? | Good | Data are collected with temporal information on when maltreatment occurred and can therefore be precisely aggregated according to a specified time interval (e.g. by calendar year) |
|  |  | Satisfactory | Data are collected with temporal information on when a case was recorded and can therefore be precisely aggregated according to a specified time interval (e.g. by calendar year) |
|  |  | Problematic | Data did not contain temporal information and therefore cannot be aggregated according to a specified time interval |
| Interpretability | Are the data easy to understand & extract? | Good | The data is stored / published in an accessible and easily understandable way, where the data it is straightforward to extract and interpret |
|  |  | Satisfactory | The data may be extracted and understood after studying relevant information which is provided in the same publications (e.g. contents pages) |
|  |  | Problematic | The data are presented in a complicated format making it difficult to interpret and extract |

**S2B Table. Quality assessment of included datasets.**

| **Dataset** | **Representativeness** | **Missingness** | **Accuracy** | **Temporal consistency** | **Validity** | **Definitions** | **Timeliness** | **Interpretability** |
| --- | --- | --- | --- | --- | --- | --- | --- | --- |
| Child Protection Statistics | Good | Satisfactory | Problematic | Problematic | Good | Good | Satisfactory | Good |
| Children In Care statistics | Good | Problematic | Problematic | Problematic | Good | Good | Satisfactory | Satisfactory |
| Criminal Statistics | Good | Satisfactory | Satisfactory | Problematic | Good | Good | Satisfactory | Problematic |
| Homicide Index | Satisfactory | Good | Good | Problematic | Good | Good | Satisfactory | Good |
| Mortality Statistics | Satisfactory | Good | Good | Problematic | Good | Good | Satisfactory | Satisfactory |
| NSPCC Statistics | Satisfactory | Good | Good | Problematic | Good | Satisfactory | Satisfactory | Good |
