# Supplementary material for: iCoverT: A rich data source on the incidence of child maltreatment over time in England and Wales
Source: PLoS One. 2018 Aug 27;13(8):e0201223. doi: 10.1371/journal.pone.0201223 (PMC6110478; doi:10.1371/journal.pone.0201223)
Supplement: S3 Table — (DOCX) [file pone.0201223.s003.docx]

**S3 Table. Excluded and truncated data variables with reason(s) for exclusion.**

| **Dataset** | **Data variables** | **Excluded or truncated** | **Reason for exclusion** |
| --- | --- | --- | --- |
| Criminal Statistics | Police-recorded data (e.g., “Offences known to the police”) | Excluded | **Unresolvable temporal consistency problems**  In 1998, changes to the HOCR clarified that the recording of crimes by police should be based on the number victims rather than recording the occurrence of the offence^b^. In 2002, the NCRS introduced changes to bring about a more victim-centred approach and increase consistency between forces. The NCRS resulted in around a 23% increase in the number of police-recorded incidences of minor violent offences^b^, affecting offences against children. As a result, police-recorded minor violent offences were excluded due problematic temporal consistency |
|  | Data relating to the offence Gross indecency with a child | Excluded | **Unresolvable temporal consistency problems**  Sexual Offences Act (2003), enacted May 2004, repealed the offence of Gross indecency with a child. After 2004 only historic offences were prosecuted, rendering Gross indecency with a child data pre- and post-2004 incomparable |
| Homicide Index | Homicide Index data pre-1977 | Truncated | **Unresolvable accuracy problems**  Prior to 1977, Homicide Index data are held in a mix of electronic and paper formats. Following a consultation with the Home Office, data pre-1977 were not considered to be accurate |
| NSPCC Statistics | Childline data | Excluded | **Unresolvable temporal consistency problems**  Although Childline was founded in 1986, contact with NSPCC’s Duty Information Specialist identified that due to changes in recording, data pre- and post- 2009/10 may not be compared |
|  | Helpline data | Excluded | **Unresolvable temporal consistency problems**  Contact with NSPCC’s Duty Information Specialist identified that due to changes in recording, data pre- and post- 2007/08 may not be compared |
|  | Data relating to caseload and number of persons helped by the NSPCC pre-1889/90 | Truncated | **Unresolvable temporal consistency problems**  The NSPCC national society was founded in 1889. Data for the five years previously consisted of aggregate data from local societies, which were not comparable to the national figures from 1889/90 onwards |

HOCR = Home Office Counting Rules.

NCRS = National Crime Recording Standard.

^a^ Povey D, Prime J. Recorded Crime Statistics, England and Wales, April 1998 to March 1999. 1999.

^b^ Simmons J, Legg C, Hosking R. National Crime Recording Standard (NCRS): an analysis of the impact on recorded crime. 2003.
