# Supplementary material for: iCoverT: A rich data source on the incidence of child maltreatment over time in England and Wales
Source: PLoS One. 2018 Aug 27;13(8):e0201223. doi: 10.1371/journal.pone.0201223 (PMC6110478; doi:10.1371/journal.pone.0201223)
Supplement: S4 Table — (DOCX) [file pone.0201223.s004.docx]

**S4 Table. Summary of changes over time, data linkage strategies and dummy variable recommendations.**

| **Database** | **Changes over time** | **Data linkage strategy** | **Dummy variable^a^** |
| --- | --- | --- | --- |
| Child Protection Statistics | **Data collection change**  The format of the data collection method changed in 2010. Prior to 2010, the data were collected from local authorities via aggregate CPR (1-3) returns. From 2010 onwards, the data were collected via the children in need census (CIN). The two data collection methods are broadly similar but changed the emphasis from national to child level data | **Strategy E: No substantial effect**  This data collection change resulted in some cases being reported multiple times as new information was reporting on already open cases. However, this issue has subsequently resolved, and data visualisations indicate that there were no abrupt increases (beyond the typical long-term trend), which suggests that this only marginally affected aggregate figures | Recommended |
|  | **Source change**  In 2003, the Department of Education subsumes the responsibility from Department of Health for publishing England child protection statistics | **Strategy C: Matched equivalent items**  From 2003 onwards, equivalent Department of Education publications were identified and matched | Not recommended |
|  | **Data change**  The categories of abuse changed over time. These changes included a shift from recording only pure categories of abuse and specific mixed categories of abuse to recording “mixed categories” of abuse which may count a child more than once from 2002 onwards | **Strategy A: Derived equivalent data**  For data prior to 2002 and Welsh data, we derived mixed categories by adding together relevant pure categories and specific mixed categories of abuse. The equivalence of these figures was checked using the overlapping periods | Not recommended |
|  | **Data change**  From October 1991 the abuse category “grave concern” was discontinued as it was not recommended in Working Together guidance under the Children Act (1989) issued by the Department of Health and Welsh Office in 1991 | **Strategy E: No substantial effect**  The category of “grave concern” was gradually discontinued over a 3-year period, therefore the data were not abruptly, substantially affected | Recommended |
|  | **Terminology change**  Some terminology changed over the years, for example the term “children on the register” later became “children subject to child protection plan” in 2007 | **Strategy C: Matched equivalent items**  We identified any changes in the language used and matched equivalent terms across years | Not recommended |
| Children In Care Statistics | **Data collection change**  Up until 1998, data on children in the care of local authorities were derived from SSDA903 returns. For this period, the SSDA903 were completed and submitted by local authorities for all children in their care. However, from 1998 to 2003 the SSDA903 only covered one third sample of children in care, those with a day of birth divisible by three. A new return was also introduced, called the CLA100, which was intended to provide provisional national figures (a fast track method of producing estimates). From 2004, the CLA100 return was discontinued and the SSDA903 reverted to collecting data form all children, and also became web-based | **Strategy D: Data prioritisation**  We investigated the differences between the SSDA903 and CLA100 data collection methods. The items on both returns were designed to be comparable, as a result there were little differences between them. However, we found that the CLA100 produced provisional, less accurate and detailed figures, than the SSDA903. We prioritised addressed the data collection changes by prioritising figures derived from SSDA903 returns for all available years, including the 1998 to 2003 period where national figures were derived from a one third sample | Recommended |
|  | **Source change**  The Home Office and then Department of Health (England) collects data from all local authorities in England and Wales up until 31 March 1991. From 1 April 1991, the Department of Health collected data from local authorities in England only, and the Welsh Office becomes responsible for collecting data from local authorities in Wales | **Combined strategies A, C & D**  We extracted from Children in care England and Wales reports (combined figures) up until 1991. From 1992 onwards, data for England and for Wales were extracted from separate reports and then added together | Recommended |
|  | **Source change**  In 2003, the Department of Education subsumes the responsibility from Department of Health for publishing England child in care statistics | **Strategy C: Matched equivalent items**  From 2003 onwards, equivalent Department of Education publications were identified and matched | Not recommended |
|  | **Time interval change**  From 1949 to 1954, Children In Care data were collected for a 12-month period ending 30 November. From 1955/56 onwards these 12-month period changed to ending 31 March | **Strategy E: No substantial effect**  As data were still consistently collected every 12 months, this change was not considered to have a substantial effect on the data | Recommended |
|  | **Terminology change**  When the Children Act 1989 came into force in October 1991 the Children In Care data were archived and a newly re-designed “Children Looked After” dataset was introduced | **Strategy C: Matched equivalent items**  Prior to October 1991, children were referred to as being ‘in care’ when the Children Act (1989) came into force in 1991 children in care of local authorities were referred to as being “looked after”. We found the change in terminology to effect labels only, as the data collection method and data were otherwise equivalent. We matched up data variables referring to children in care prior to 1991 to children looked after from 1991 onwards | Not recommended |
| Criminal Statistics | **Structural change**  Following the enactment of the Courts Act (1971) in January 1972, Courts of Assizes and Quarter Sessions were replaced by a single Crown Court with the power to sit anywhere in England and Wales, and deals with all indictable offences | **Strategy A: Derived equivalent data**  Before 1972, we added together figures from quarter sessions and courts of assizes to get a single figure that was comparable to the Crown Court from 1972 onwards | Recommended |
|  | **Organisational change**  In July 1978 Part III of the Criminal Law Act (1977) was implemented resulting in a re-classification of offences to: (i) offences triable only on indictable at the Crown Court, (ii) offences triable-either-way at the Crown or magistrates’ courts, and (iii) offences triable only summarily at magistrates’ courts. *Cruelty to and neglect of children* was re-classified as an offence that is triable-either-way from 1979 onwards, whereas previously two *Cruelty to and neglect of children* offences, offence 11 and 109, were tried as an indicatable and summary offence, respectively | **Strategy A: Derived equivalent data**  Before 1979, offence numbers 11 and 109 were added together to form a single total to represent *Cruelty to and neglect of children*. This figure was comparable to the single figure published from 1979 onwards | Recommended |
|  | **Source change**  Until 2007, the Home Office was responsible for publishing the Criminal Statistics dataset. From May 2007, the newly formed Ministry of Justice subsumed the responsibility | **Strategy C: Matched equivalent items**  The equivalent data were identified and matched in the pre-2007 Home Office and post-2007 Ministry of Justice publications | Recommended |
|  | **Data change**  Until 1991, the number of persons cautioned were not published in the annual reports. However, from 1992, “persons cautioned” were not only introduced, but were also added to the number of persons found guilty. Where reports had previously presented “persons found guilty” they now presented “persons cautioned and found guilty” | **Strategy A: Derived equivalent data**  In order to calculate the number of “persons found guilty” from 1992 onwards, the number of “persons cautioned” was subtracted from “persons cautioned and found guilty” | Not recommended |
|  | **Definition change**  Legal definition changes for the offence *Cruelty to and neglect of children* include: Children & Young Person Act (1933), enacted November 1933, changed age of male victims from under 14 to under 16 and consolidated previous legislation to defining the Cruelty to children. Children Act (1989), enacted October 1991, amended the words “has the custody, charge or care of” to “has responsibility for”. Serious Crime Act (2015), enacted March 2015, makes explicit that cruelty includes cruelty causing psychological suffering | **Strategy E: No substantial effect**  It was determined that the legal definition was not drastically altered over time and therefore the effect of the definition change was not determined to be substantial | Recommended |
|  | **Definition change**  Legal definition changes for the offences *Unlawful sexual intercourse with a girl under 13* and *Unlawful sexual intercourse with a girl between 13 and 16*: “Defilement of girls” changes to unlawful sexual intercourse with Sexual Offences Act (1956), enacted January 1957. Sexual Offences Act (2003), enacted May 2004, repealed section 5 & 6 of the Sexual Offences Act (1956) and redefined these sexual offences to “Sexual activity” with a child, rather than only girls. These offences were further categorised in terms of harm (1,2,3) and culpability (A, B) | **Strategy C: Matched equivalent items**  Following investigations, it was determined that post-2004 the offence numbers 21.1 and 22.1 (i.e. harm 1) including culpability A and B best represented the offences *Unlawful sexual intercourse with a girl under 13* and *Unlawful sexual intercourse with a girl between 13 and 16*, respectively | Recommended |
|  | **Terminology change**  Published reports inconsistently used the terms “found guilty” and “convicted” | **Strategy A: Derived equivalent data**  For each inconsistent time period (9 identified), the different uses of “found guilty” and “convicted” were outlined. By understanding the criminal system, and changes to this over time, we used sub-divisions within these categories (e.g. “proceeded against”, “withdrawn/dismissed”) to establish a unified definition and corresponding calculations, to establish temporally consistent figures for “found guilty” and “convicted” | Not recommended |
| Homicide Index | **Recording change**  Changes to the recording rules HOCR in 1998 and NCRS in 2002 aimed to make police recorded crime more consistent and victim-oriented | **Strategy E: No substantial effect**  Although police recorded crime were subject to recording changes, these changes did not affect the recording of homicides. This is because the recording rules emphasised a victim-oriented approach and victims may not report incidents in the case of homicides. The number of homicides were therefore not affected by the recording change^b^ | Not recommended |
|  | **Definition change**  The Corporate Manslaughter and Corporate Homicide Act (2007), which came into force on April 2008, meant that definitions of homicide now included corporate manslaughter | **Strategy E: No substantial effect**  Data visualisations indicated that the number of child homicides decreased from 2008 to 2009, indicating that the broadening of the homicide definition did not significantly increase the number of child homicide victims | Not recommended |
|  | **Time interval change**  From 1977 to 1997, police-recorded crime was reported for 12-month periods ending 31 December. In 1997/98 this 12-month period changed to ending 31 March | **Strategy E: No substantial effect**  As data were still consistently collected every 12 months, this change was not considered to have a substantial effect on the data | Recommended |
|  | **Data change**  From 1977 to 2016 the age categories varied over time. For example, from 1977 to 1992 the category “under 5 years” was collected and from 1993 onwards this was divided into two categories of “under 1 year” and “from 1 to 4 years” | **Strategy B: Broader category**  We extracted broader age categories that could be matched over time. For example, “under 5 years” which involved adding together “under 1 year” and “from 1 to 4 years” from 1993 onwards | Not recommended |
| Mortality Statistics | **Recording change**  The process of implementing ICD codes to cause of death changed over years, first from manual to automatic coding and then changes to the software used (e.g. introduction of IRIS software in January 2014) | **Strategy E: No substantial effect**  We studied the potential impact of such changes by examining existing research. The Office for National Statistics have carried out a handful of studies to examine the impact of changes to the coding process. These studies found that such change had marginal effects. For example, the introduction of the IRIS software only affected 95% of coded deaths^c^. As a result, we determined the recording change to have no substantial effect | Not recommended |
|  | **Data change**  From 1858 to 2016 the classification of cause of death changed according to evolving ICD codes, therefore codes were often inconsistent across time periods where the ICD version had been modified | **Strategy B: Broader category**  Following and extending previous methodological approaches^d^, we classified causes of death according to three broader categories: Homicides/assault; Undetermined intent; and Unknown cause. Full details specified in the Homicide Index Data guide | Recommended |
|  | **Data change**  From 1858 to 2016, the age categories varied over time | **Strategy B: Broader category**  We extracted broader age categories which were consistent over time, including a category of 75+ to deal with notable changes to the average life expectancy | Not recommended |
| NSPCC Statistics | **Geographical change**  From 1890 to 1907 the NSPCC statistics covered England, Wales, Ireland and Scotland. However, NSPCC stopped working in Scotland in July 1907, therefore from 1908 onwards the statistics no longer covered Scotland. Up to 1956 the NSPCC Statistics covered England, Wales and Ireland. However, in March 1956 the ISPPCC was founded and the NSPCC only worked in Northern Ireland from 1957 onwards | **Strategy E: No substantial effect**  We were unable to determine the true effect of these geographical changes, but after consultation with a NSPCC Duty Information Specialist, we determined that the data were still comparable over time | Recommended |
|  | **Time interval change**  From 1890 to 1946 data were collected for 12-month periods ending 31 March. In 1947, this changed to a 12-month period ending 28 or 29 February, in 1970 this changed to ending 31 December, and in 1974 to ending 30 September | **Strategy E: No substantial effect**  As data were still consistently collected every 12 months, this change was not considered to have a substantial effect on the data | Recommended |
|  | **Data change**  The data categories for age changed over time. For example. From 1951 to 1978, children aged under 5 were categorised as “Under 2 years” and “2 to under 5 years”. However, from 1979 onwards, there was only the single category of “Under 5 years”. In addition, the age categories underwent five separate category changes | **Strategy B: Broader category**  We extracted and calculated the broader age categories of “Under 5 years” and “5 years and over”, these categories were consistent over time | Not recommended |
|  | **Data change**  The data categories for who referred the NSPCC case changed over time. For example, from 1921 to 1969 the categories consisted of: the general public (including parents & relatives), police officials, school officials, other officials, and NSPCC society officials. However, from 1970 onwards, the changed to the categories: officials, general public (excluding parents & relatives), parents, and other relatives | **Strategy B: Broader category**  We extracted and calculated two broader categories of the general public (including parents and relatives) and officials (including police, school, other, and NSPCC) these categories were consistent over time | Not recommended |
|  | **Data change**  The data categories for the type of NSPCC case changed over time. Most notably, the type of offence of “corruption of morals” was no longer reported from 1953, instead two new categories of “moral danger” and “immoral offences” | **Strategy A: Derived equivalent data**  Following consultation with a NSPCC Duty Information Specialist, we identified that corruption of morals pre-1953 was equivalent to the sum of “moral danger” and “immoral offences” post-1953 | Not recommended |

SSDA903 = Social Services Department Annual statistical return.

CLA100 = Children Looked After annual statistical return.

HOCR = Home Office Counting Rules.

NCRS = National Crime Recording Standard.

ICD = Internal Classification of Diseases.

^a^ A dummy variable is a binary variable representing temporal change. Dummy variables are created by identifying the year of change: years before this change are coded as 0, and the year of change and years after change are coded as 1.

^b^ Povey D, Prime J. Recorded Crime Statistics, England and Wales, April 1998 to March 1999. 1999.

^c^ Office for National Statistics. Impact of the Implementation of IRIS Software for ICD-10 Cause of Death Coding on Mortality Statistics, England and Wales [Internet]. 2014. Available from: <http://www.ons.gov.uk/ons/dcp171778_373602.pdf>

^d^ Sidebotham P, Atkins B, Hutton JL. Changes in rates of violent child deaths in England and Wales between 1974 and 2008: an analysis of national mortality data. Arch Dis Child. 2012;97(3):193–9.
