# Supplementary material for: iCoverT: A rich data source on the incidence of child maltreatment over time in England and Wales
Source: PLoS One. 2018 Aug 27;13(8):e0201223. doi: 10.1371/journal.pone.0201223 (PMC6110478; doi:10.1371/journal.pone.0201223)
Supplement: S5 Table — Note that full details of data adjustments for each database are detailed in the Data dictionaries. (DOCX) [file pone.0201223.s005.docx]

**S5 Table. Summary of data adjustments.**

| **Database** | **Data imputations** | **Data idiosyncrasies** | **12-month period** | **Precision (rounding)** |
| --- | --- | --- | --- | --- |
| Child Protection Statistics | n/a | Welsh data relating to the age and gender of children on the child protection register for the year 1992, and the gender of children who were de-registered in England for the years 2010 to 2012, were provisional. Provisional data consisted of raw local authority data which had not been adjusted to account for missing data, deriving national estimates. To address this and derive national estimates, we adjusted the data via the following two-steps:   1. Summed the provisional figures to calculate the raw data total only (including missing data) 2. For each year, calculated the ratio between raw data total and given national estimates $Ratio =\frac{given national estimate}{raw data total}$ 3. Multiplied the raw data by this ratio to derive national estimates and adjust for missing data | n/a | Over time, children in care data were inconsistently reported to the nearest 5, 10 or 100. All data were therefore rounded to the highest common level of precision, which was typically 5 or 10 for Welsh data, and 100 for English data. These rounding adjustments may result in some rounding errors |
| Children In Care Statistics | Missing data for the number of new children in care during the years 1952 to 1976. However, from 1952 to 1982 data on the number of new care periods in care are reported. Care periods are a period in which a child is continuously in care.We therefore carried out the following two-step ad-hoc data imputation to derive data for the missing years 1952 to 1976:   1. For 6 years (1977-1982) data for new children and new care periods were both reported. We therefore established the average ratio between new children and new care periods from 1977 to 1982 $Average ratio= \sum\frac{new children}{new care periods}$ 2. This average ratio was then used as a multiplicative factor and multiplied with the number of new care periods for each year from 1952 to 1976 | During the year 1970/71, amendments set out in the Children & Young Person Act (1969) para. 7(2) of Schedule 4 stated that children who would have been subject to an approved school order, or to supervision following release from approved school, on the 31 Dec 1970 were now deemed to be in care. As a result, there was an abrupt increase of children in care. To adjust for this artificial increase, the number of children affected by this amendment was subtracted from the appropriate figures | Data for the year 1991/92 figure only covered 6 months (October 1991 to March 1992). All 1991/92 figures were therefore multiplied by 2 to account for the missing 6 months. All resulting figures were rounded to the nearest integer | Over time, children in care data were inconsistently reported to the nearest 10 or 100. All data were therefore rounded to the highest common level of precision, which was typically 10 for Welsh data, and 100 for English data. These rounding adjustments may result in some rounding errors |
| Mortality Statistics | n/a | For the year 1858, only a summed figure for male and female homicide victims aged 15 to 24 was given, instead of a two category age breakdown of victims aged 15 to 19 and 20 to 24. By calculating a 3-year average for the ratio between victims aged 15 to 19 and aged 20 to 24. Data for 1858 were then multiplied by these ratios to approximate figures for the age categories 15 to 19 and 10 to 24. All resulting figures were rounded to the nearest integer | n/a | n/a |
| NSPCC Statistics | n/a | For the years 1915 to 1920, a summed total of the number of war enquiries for the number of NSPCC cases and children helped was given. We divided these figures by the number of years, 5, and added them to non-war enquiries for each respective year.  For the year 1968/69 the age of children helped by the NSPCC was only given as a percentage of the total number of children. We calculated these percentages and entered them as raw data. All resulting figures were rounded to the nearest integer | Data for the year 1946/47 only covered 11 months (April 1946 to February 1947). All 1946/1947 figures were therefore divided by 11 and multiplied by 12 to derive 12-month estimates. Data for the year 1973/74 only covered 9 months (January 1974 to 30 September 1974). All 1973/74 figures were therefore divided by 9 and multiplied by 12 to derive 12-month estimates. All resulting figures were rounded to the nearest integer | n/a |

Note that full details of data adjustments for each database are detailed in the Data dictionaries.
